# BREAKING BAD NEWS IN A NEONATAL INTENSIVE CARE: THE PARENT’S EVALUATION

**DOI:** 10.1590/1984-0462/2020/38/2019092

**Published:** 2020-06-05

**Authors:** Ligia Marçola, Ivete Zoboli, Rita Tiziana Verardo Polastrini, Silvia Maria Macedo de Barbosa

**Affiliations:** a Universidade de São Paulo, São Paulo, SP, Brazil.

**Keywords:** Infant, newborn, Congenital abnormalities, Health communication, Intensive care units, neonatal, Recém-nascido, Anormalidades congênitas, Comunicação em saúde, Unidades de terapia intensiva neonatal

## Abstract

**Objective:**

To describe the reports of parents of newborns (NB) with congenital malformations hospitalized in a Neonatal Intensive Care Unit (NICU) who received bad news, in order to identify the issues related to the perception of bad news given adequately or inadequately.

**Methods:**

A cross-sectional study was conducted from January to October 2018, in which parents of newborns with congenital malformations hospitalized in NICUs were interviewed at visiting hours, according to inclusion criteria. The questionnaire had semi-structured questions related to reception of bad news. Analysis of the data was descriptive.

**Results:**

28 mothers and two fathers were interviewed and 16 (53.3%) reported having had at least one bad news in the NICU. Of those, 10 (62.5%) considered appropriate the way in which the news was given. The justifications were: sincerity of the professional, delicacy to give the news, giving hope to the family, use of appropriate words and demonstration of caring about the newborn. Six participants (37.5%) considered inadequate the way of breaking bad news. The reasons were: unpreparedness and lack of knowledge about the child’s case, use of difficult language, haste or anxiety and discouragement of family hope. Most of the news was given by a professional alone, often by a medical resident.

**Conclusions:**

The communication of bad news was considered adequate by the parents, although this perception was not unanimous. This study, therefore, indicates that it is necessary to improve the communication of bad news in this NICU. Training professionals can assist in this process.

## RESUMO

**Objetivo:**

Descrever os relatos dos pais de recém-nascidos (RNs) com malformações congênitas internados em uma Unidade de Terapia Intensiva Neonatal (UTIN) a respeito de como receberam as más notícias, buscando identificar as questões relacionadas à percepção de uma má notícia fornecida de forma adequada ou inadequada.

**Métodos:**

Realizou-se estudo transversal de janeiro a outubro de 2018, no qual se entrevistaram pais de RNs portadores de malformações congênitas internados em UTIN selecionados segundo critérios de inclusão e presentes em horário de visita. O questionário utilizado tinha questões semiestruturadas pertinentes ao recebimento de más notícias. A análise foi descritiva.

**Resultados:**

Entrevistaram-se 28 mães e dois pais, dos quais 16 (53,3%) apontaram ter tido pelo menos uma má notícia na UTIN. Destes, 10 (62,5%) consideraram adequada a maneira de dar essa notícia. As justificativas foram: sinceridade do profissional, delicadeza para dar a notícia, dar esperança à família, uso de palavras adequadas e cuidado demonstrado com o RN. Seis participantes (37,5%) avaliaram como inadequada a comunicação de más notícias. Motivos foram despreparo e falta de conhecimento, uso de linguagem difícil, pressa ou ansiedade e desencorajamento de esperanças da família. A maior parte das notícias foi dada por um profissional sozinho, muitas vezes por um médico residente.

**Conclusões:**

A percepção da comunicação de más notícias foi considerada adequada por parte dos pais, embora não tenha sido unânime. Este estudo aponta ser necessário melhorar a comunicação dessas notícias na UTIN analisada. O treinamento dos profissionais, nesse sentido, pode auxiliar nesse processo.

## INTRODUCTION

The need for specialized assistance in Semi-Intensive Care Units or Neonatal Intensive Care Units (NICUs) brings a lot of anxiety, anguish and uncertainty to newborns’ (NB) families, who, during pregnancy, expected a healthy child.^1^ ^,^ ^2^ ^,^ ^3^ ^,^ ^4^ ^,^ ^5^ Frequent and adequate communication between the team and family members is essential to ensure understanding about what is happening with the newborn^2^ and to reduce this stress.^3^ ^,^ ^4^

The NICU environment is full of sounds, words, images and odors that can cause discomfort or strangeness to families, especially those that are newly admitted.^1^ Often, in contrast to the large amount of structural resources and technology available, little attention is paid to communication.^2^ ^,^ ^6^ In the United Kingdom and other European countries, the main complaint of families has been related to this issue: the lack of appropriate communication and information given regularly.^2^ ^,^ ^3^ ^,^ ^6^ There are few studies carried out in Latin American countries about communication in NICUs,^7^ ^,^ ^8^ ^,^ ^9^ and those that exist also show the need to improve and adapt this aspect of care.

Communication is defined as the transmission of information between people, which can be done verbally and non-verbally. More than that, it often means sharing information, and offering emotional support. Thus, empathy is essential for this communication to be appropriate.^3^ ^,^ ^5^ ^,^ ^10^ As far as being informed about their children, parents in the NICU often want to share their anxieties and feelings, and be heard.^1^ ^,^ ^4^ ^,^ ^10^ In addition, communication must be considered a two-way street. Ways of understanding the information that one seeks to transmit also depends on the sociocultural and personal context of the recipient. Therefore, in the communication process, both parties need to understand and support each other. ^9^

In this context, giving bad news is a complex and challenging task for health professionals, and receiving it is very difficult for the family. Bad news can be understood as any information that affects the individual’s perception of his or her future, which is considered negative by the recipient.^9^ ^,^ ^11^ The family’s first experience in which this news is given greatly influences their way of looking at the problem and dealing with it later on. As such, it is very important that the professional involved is aware of how the bad news is given.^9^ ^,^ ^12^ ^,^ ^13^

There are many protocols and recommendations in the literature that, when put into practice, have been shown to improve the family’s perception on when to receive bad news, but even so, professionals often feel uncomfortable with the family’s emotional reactions, and feel guilty and frustrated. Studies with doctors of varying degrees of experience show that they lack training and education in this area.^9^ ^,^ ^11^

Since there has been little explored on this subject in Brazil and Latin America, and considering the differences and peculiarities of European and North American services, it is essential to evaluate communication and how bad news is given, so that interventions are well thought out, as needed.

The objective of this research was to describe the reports of parents of newborns with congenital malformations admitted to an NICU regarding how they received bad news. Ultimately, it sought to identify issues related to the perception of bad news given appropriately or not.

## METHOD

A prospective cross-sectional study was conducted in which parents of newborns with congenital malformations admitted from January 3 to October 24, 2018 at the Instituto da Criança, in the neonatal intensive care center-2 (CTIN-2) were interviewed. Participants were selected according to inclusion criteria and interviewed during visiting hours.

The external NICU where the study took place is part of the Hospital das Clínicas (HC) Complex of the Faculty of Medicine of the Universidade de São Paulo (FM-USP), in São Paulo. It is located in a tertiary university hospital that receives neonates from all over Brazil. These neonates are carriers of malformations, such as diaphragmatic hernias, gastroschisis, omphalocele, among others. Neonatology resident doctors, preceptors and other assistant doctors all work in this place.

The inclusion criteria were: parents accompanying newborns with major congenital malformations admitted to the CTIN-2 for more than seven days, and who signed the free and informed consent form. Exclusion criteria were family members who were not fluent in Portuguese and/or under 18 years old.

The interviews were done by the researcher or by a previously trained assistant, usually once a week, almost always in the morning. To prepare the questionnaire (available with authors), a literature review was made^2^ ^,^ ^3^ ^,^ ^4^ ^,^ ^8^ ^,^ ^9^ ^,^ ^11^ and there was discussion in a multidisciplinary team. Thus, characteristics of parents were raised that could influence their opinion in relation to the way of giving bad news -- age, education and previous experiences in the Intensive Care Unit (ICU), among others. It was also concerned with describing the conditions in which the malformation was diagnosed and followed up (prenatal care and place where it was performed), as well as the place of birth. We analyzed: length of hospital stay, age and education of family members, previous experiences in the NICU, place of prenatal care and birth, participation in the Comprehensive Support Group for Pregnant Women and Families of Fetuses with Malformations (*Grupo de Apoio Integral a Gestantes e Familiares de Fetos com Malformação* - GAI), which tracks the cases of multiple malformations, severe malformations, and those with a high risk of perinatal death at the HC of the FM-USP. Family members were asked if they had received bad news during that hospitalization, who gave the news, whether they had found the way of giving this bad news appropriate, and why. Question number 18 of the interview - why to consider the news given appropriately or not - was open; for analysis, the responses were later categorized into items.

The research was initiated after approval by the Research Ethics Committee of the department involved, the institution’s Research Ethics Committee and the National Research Ethics Committee. Patients whose medical records were included in the study and their parents were not and will not be identified in any publication or research material.

## RESULTS

A total of 30 companions were interviewed. The average length of stay until the day of the interview was 27 days (range 7-153 days, standard deviation - SD=32 days). A total of 28 mothers and two fathers participated, aged 18 to 41 years old, with an average age of 29 years old and a SD of 6 years. Of the 30 participants, 13% had completed elementary school, 50% had completed high school, 27% had completed higher education and 10% had postgraduate degrees. A total of 22 (73%) had other children. Six interviewees had already had the experience of accompanying a child in the NICU.

Of the 30 cases of malformed newborns, 80% had a prenatal diagnosis. In relation to prenatal care, 87% were performed in a high-risk outpatient clinic - 10% had been monitored by GAI and 80% were born at the HC Complex of the FM-USP.

In the 30 interviews, 16 participants reported having received at least one piece of bad news in the unit. Tables 1 and 2 show the sample characterization data. Of these 16 parents, 10 (62.5%) considered the way of giving this news adequate. In this case, the professional’s sincerity/honesty was mentioned in 50% of the cases; the delicacy/the way of breaking the news, was mentioned by 60%; and respect or hope given to the family was mentioned by 40%. A family member mentioned the clarity/use of appropriate words when giving the news, and one spoke about the care that the health professional demonstrated in having with the NB.

**Table 1:** Characterization of the sample of companions (mother or father) who received the bad news.

		n	%
Age	≤25 years	12	40
	> 25 years	18	60
Education level	Completed higher education	11	37
	No higher education	19	63
Prenatal location	BHU	22	73
	High-risk outpatient clinic	26	87
	Health insurance/private	3	10

BHU: Basic Health Unit

**Table 2:** Characterization of the sample of companions and situations related to bad news.

	Yes		No	
	n	%	n	%
Prenatal diagnosis of MF	24	80	6	20
Born at HC of the FM-USP	24	80	6	20
Participation in GAI	3	10	27	90
First child	8	27	22	73
Previous experience in NICU	6	20	24	80
Received bad news	16	53	14	47
The way of giving the news was considered to be appropriate	10	62.5	6	37.5

MF: malformations; HC: Hospital das Clínicas; FM-USP: Faculty of Medicine, Universidade de São Paulo; GAI: Comprehensive Support Group for Pregnant Women and Relatives of Fetuses with Malformation (*Grupo de Apoio Integral a Gestantes e Familiares de Fetos com Malformação*); NICU: Neonate intensive care unit.

Of the 16 parents, six (37.5%) rated the bad news as given inappropriately. In this situation, the reasons cited were the health professional’s lack of preparation and lack of knowledge about the child’s case (2), the use of words and language that were difficult to understand (2), haste or anxiety of the person(s) involved (1), and crushing the family’s hopes (1). One of the interviewed mothers said that she had heard the news inadvertently, in a conversation between two staff members in the unit, who did not see her nearby. The interviewees’ statements are transcribed in Tables 1 and 2.

**Chart 1:** Interviewees’ statements: bad news given inappropriately.

*“The doctor seemed very nervous, she looked at her watch, she looked around, she didn’t pay much attention.”*
*“The doctor was not at all delicate, she said all of the difficult things and left me there, alone, worried and without understanding.”*
*“I overheard. In fact, no one came to speak to me directly.”*
*“It seemed like she didn’t know everything to be able to explain it to me, you know. And it took away all my hopes saying that there was no way.”*
*“Very unprepared, they don’t know how to talk properly.”*
*“It was not very good, no. The doctor didn’t seem to know what was happening to my son.”*

**Chart 2:** Interviewees’ statements: bad news given appropriately.

*“She explained everything to me and asked me to have faith.”*
*“She started talking little by little, tried to calm me down, you know.”*
*“The surgeon even showed me a video of my son’s surgery, it was easy to understand.”*
*“It was very honest, they didn’t hide anything, but it was very delicate, and they calmed me down afterwards.”*
*“They gave me all of the correct information, but without being scary, you know.”*
*“They knew how to empathize, they comforted me.”*
*“The doctor was very delicate, but she didn’t hide anything.”*
*“The phono [audiologist] was talking little by little so I wouldn’t get scared; they showed that my daughter had been assisted. They had tried, but there was no way, she was going to need the gastrostomy.”*
*“I was worried when the resident told me that she might need to intubate [my daughter], but then she gave me a lot of hope that it might not be necessary.”*
*“They were very polite, some really have a way of talking, you know. They answered all of my questions.”*
*“The resident was very clear in the explanations, but she was very gentle. She spoke slowly, with skill.”*

Sixty-nine percent of the time (n = 11) the neonatology resident doctor was present during the transmission of this bad news; 64% of the time (n = 7) he broke this news alone. In one case he was with the nurse. And only in 27% (n = 3) of the time, he was with a more experienced physician (assistant). The presence of the assistant neonatologist took place in only 37.5% of the cases (n = 6), once alone, three times with the resident, in one case with the speech therapist, and in one case with the surgeon. Nursing and speech therapists were mentioned only once in each case. In one situation, the NB’s father was the bearer of bad news.

## DISCUSSION

This study identified a situation similar to that of NICUs and pediatricians in other places in Brazil, the United States and Europe, in which many parents are not satisfied with the way they receive bad news. Complaints about communication are very similar: difficult language;^4^ ^,^ ^9^ ^,^ ^11^ inadequate professional posture: demonstration of haste/nervousness,^3^ ^,^ ^11^ aggressiveness;^4^ lack of empathy/delicacy/affection in the way of speaking;^4^ ^,^ ^9^ ^,^ ^11^ ^,^ ^12^ not giving or discouraging the family’s hope.^9^ Among the satisfied family members, as observed in the literature, the delicacy of a careful, but honest and clear conversation, which did not take away the family’s hope, was mentioned.

It is worth noting that bad news in general is given in this NICU by individual professionals. In only 36% of the times at least two professionals were together during this task. Perhaps because they consider it to be a less important task or even have a defensive attitude with regard to the challenge of giving bad news, preceptors often delegate it to the resident, and the resident is almost always alone. The residents haven’t been specifically trained for it and are naturally inexperienced. Their fears, insecurities, limited time and lack of support from supervisors are barriers to effective communication and adequate interaction with family members.^14^ Studies show that most doctors in training do not feel safe to give bad news alone. Negative experiences can even be responsible for negatively shaping this professional’s way of giving bad news, protecting themselves with the use of inappropriate language and lack of empathy.^15^ On the other hand, even experienced doctors also feel that they do not have enough education or preparation to deal with this difficult news. Most would like to be able to have more training.^14^ ^,^ ^15^ It is known that the professional training is effective in improving relationships with families and increasing satisfaction with the way the news is given.^10^ ^,^ ^13^ ^,^ ^14^

In this sense, the use of protocols in these trainings can be very useful, as they work as a guide to give bad news. A well-known example is the SPIKES protocol, developed and used widely in oncology, but applicable in any clinical situation when dealing with bad news. It constitutes a series of six consecutive steps and creates a favorable environment to spread news in a delicate, informative and compassionate way:

- *Setting*: refers to an appropriate place for the conversation, but also to the presence of important people for whom the news is received (professionals and family members);
- *Perception*: understand what the patient and/or family member knows about the disease;
- *Invitation*: check and respect the amount of information desired;
- *Knowledge*: passing on information gradually, using pauses and repetitions to allow the person to take it in;
- *Emotions, empathy*: showing support and understanding by welcoming the feelings provoked by the news;
- *Strategy, summary:* closing the conversation and including a care plan.

Just like SPIKES, there are other similar protocols, such as EMPATHY - which recommends dealing with Emotions in an appropriate environment *(*Meeting), first understanding the knowledge that Patient/family already has, use of Appropriate language, the patient/family’s right to the Truth and Hope, and their empowerment (Yes for empowerment) for decision making.^16^

It is interesting to note that, if the bad news communication protocols mentioned are followed, it is unlikely that a professional will be giving the news alone, as these protocols encourage the presence of other significant people for the receivers of this news. In addition, all points that were considered to be positive by the parents of the study patients will be covered during the conversation in which the bad news is given: support, empathy, delicacy, honesty.

Something that can also contribute to families’ satisfaction with communication in general and even with the way of receiving bad news is perinatal palliative care, as clearly seen in the literature.^17^ Considering that 80% of parents already received a diagnosis of their child’s malformation in the prenatal period, many could have already started the communication process at that time. Groups like GAI can help a lot in this context. They need to expand their activities, noting that, in the present study, for example, only three families had received this type of monitoring.

As limitations evidenced in the present study, we can highlight the small sample size and for convenience - only the parents present at the visit at the time of the interview. Statistical analysis was not possible due to the limited number of cases studied. Despite this, crucial questions were raised in relation to how to give bad news in this NICU and important points to be worked on: preparation for the conversation; an unhurried posture, aware of all the necessary information; speaking clearly, without medical language; delicate, but honest, and complete; and without taking away all hopes from the family. This research highlights, therefore, that there is a long way to go to improve the communication of bad news in this NICU. As noted in the literature, the training of service professionals will allow for them to follow this path to improving care.
